# FENDRR suppresses cervical cancer proliferation and invasion by targeting miR-15a/b-5p and regulating TUBA1A expression

**DOI:** 10.1186/s12935-020-01223-w

**Published:** 2020-05-06

**Authors:** Yunheng Zhu, Xiaohua Zhang, Lifeng Wang, Xiuxiang Zhu, Ziyin Xia, Ling Xu, Jun Xu

**Affiliations:** 1 Department of Obstetrics and Gynecology, Minhang Hospital, Fudan University, No.170 Xinsong Road, Minhang District, Shanghai, 201199 China; 2 Minhang District Maternal and Child Health Hospital, Shanghai, 201102 China

**Keywords:** FENDRR, Cervical cancer, miR-15a/b-5p, TUBA1A

## Abstract

**Background:**

Previous literature has revealed long non-coding RNAs (lncRNAs) are crucial regulators for cell functions and gene expression. LncRNA fetal-lethal non-coding developmental regulatory RNA (FENDRR) was reported as a biological suppressor in several types of human cancers, yet relevant mechanisms and biological effects of FENDRR with regards to cervical cancer (CC) are not explored until now.

**Methods:**

In this study, quantitative real-time polymerase chain reaction (qRT-PCR) analysis detected gene expression in tissues and cells. Gain- or loss-of-function experiments revealed the biological effects of FENDRR and miR-15a/b-5p on CC cell functions. Bioinformatics tools were used to predict the relevant genes. Mechanism experiments including RNA immunoprecipitation (RIP) assay, pull down assay and luciferase reporter assay depicted the binding situation and coexistence of indicated genes.

**Results:**

FENDRR was downregulated in CC tissues and cells, which suppressed CC progression. MiR-15a-5p and miR-15b-5p shared binding sites with FENDRR and had interaction with FENDRR. Tubulin alpha1A (TUBA1A) was downregulated in CC tissues and positively modulated by FENDRR. TUBA1A was the target of miR-15a/b-5p. TUBA1A silencing rescued the effect of FENDRR overexpression on CC cell growth and migration.

**Conclusion:**

FENDRR inhibits CC progression through upregulating TUBA1A in a miR-15a/b-5p-dependent manner.

## Background

Despite the widespread screening programs and promotion of vaccine, cervical cancer (CC) remains the third most common cancer in developing countries [1, 2]. Each year, over 260,000 women died of this disease, most of them from low-and-middle-income countries [3]. Also, the prognosis of advanced patients with CC is still dismal. The 5-year survival rate of patients in advanced CC stage is approximately as low as 15% [4]. A large set of molecular imaging biomarkers have been recognized and confirmed to predict outcome at baseline or early during cervical cancer therapy, which immensely contributes to cervical cancer diagnosis and prognosis [2]. Based on the fact that CC is still a lethal cancer for women, there is a great need and urgency to identify novel treatment targets and prognosis biomarkers.

Long non-coding RNAs (lncRNAs) are biological regulators in a wide variety of important cellular processes including proliferation, migration and epithelial-mesenchymal transition (EMT) [5, 6]. For example, lncRNA maternally expressed gene 3 (MEG3) inhibits cell proliferation and metastasis in gastric cancer [7]. LncRNA nuclear paraspeckle assembly transcript 1 (NEAT1) drives cell proliferation and EMT in breast cancer [8].

Fetal-lethal non-coding developmental regulatory RNA (FENDRR) is a new-founded lncRNA which has been reported as a tumor suppressor in different cancers including non-small lung cancer, prostate cancer, breast cancer, cholangiocarcinoma and hepatocellular carcinoma [9–12]. However, biological effects and its mechanism with respect to CC remain to be explored. Mechanistically, FENDRR can exert tumor-inhibitory functions in colon cancer through repression of Sry‑Related HMG‑BOX‑4 (SOX4) protein [13]. Moreover, FENDRR post-transcriptionally regulate Runt-related transcription factor 1 (RUNX1) by sponging miR-18a-5p [14]. In this study, we explored the functions of FENDRR in CC cell growth and migration. More importantly, we explored the downstream molecular mechanism of FENDRR in CC.

## Methods

### Clinical specimens and cell lines

The written informed consents were signed by 53 participants, and approval was acquired from the Ethics Committee of Minhang Hospital. 53 CC and adjacent non-cancer tissue samples were snap-frozen at − 80 °C in liquid nitrogen. CC cell lines including HeLa, SiHa, CaSki and C33A, as well as normal cell line Ect1-E6E7, were procured from the American type culture collection (ATCC) (Manassas, VA), incubated in the Dulbecco’s modified eagle medium (DMEM) adding the 10% fetal bovine serum (FBS) at 37 °C under 5% CO_2_.

### Quantitative real-time polymerase chain reaction (qRT-PCR)

Isolation of the total RNA from HeLa and SiHa cells were used to synthesize complementary DNA (cDNA) for conducting quantitative PCR using SYBR Green kit (TaKaRa, Shiga, Japan) on the Step-One Plus Real-Time PCR System (Applied Biosystems, Foster City, CA). RNA expression level was calculated through 2^−ΔΔCt^ method. Glyceraldehyde-3-phosphate dehydrogenase (GAPDH) and U6 small nuclear RNA (U6) were used as the corresponding internal genes for lncRNA/mRNA or microRNAs (miRNAs).

### Transfection

Confluent CC cell lines were prepared in 24-well plates for 48 h of transfection utilizing Lipofectamine 2000 (Invitrogen, Carlsbad, CA). For gene overexpression, OE/FENDRR and negative control (NC), miR-15a/b-5p mimics and miR-NC, OE/TUBA1A and NC, were produced by Genepharma (Shanghai, China). For gene silencing, miR-15a/b-5p inhibitor and miR-NC, sh-TUBA1A and sh-NC, were acquired from Genechem Company (Shanghai, China).

### CCK-8 assay

HeLa and SiHa were treated with cell counting kit 8 (CCK-8) reagents (Dojindo Molecular Technologies, Kumamoto, Japan) in the 96-well plates after transfection and detected at indicated times. Cell viability was monitored by detection of absorption at 450 nm.

### Colony formation assay

Transfected CC cell lines were placed into the 6-well plates for 2 weeks, then treated with the fixation and staining using 4% paraformaldehyde and 0.1% crystal violet.

### Flow cytometry analysis for apoptosis

Cells were reaped after transfection and fixed on ice for 1 h, followed by incubation with Annexin V-APC and 7-AAD kit in the dark. CC cells were exposed to analysis of flow cytometer.

### Transwell assays

Cell invasion or migration assay was conducted with the 24-well transwell chamber (Costar, MA) coating matrigel or not. CC cells with invasive or migratory ability were dyed with 0.1% crystal violet solution after fixing for 30 min.

### Western blot

Cellular protein sample was extracted for separation through electrophoresis on 12% sodium dodecyl sulfate polyacrylamide gel electrophoresis (SDS-PAGE). Sample on polyvinylidene fluoride (PVDF) membranes were treated with 5% nonfat milk and incubated with the specific primary antibodies (Abcam, Cambridge, MA) to E-cadherin, N-cadherin, Vimentin and GAPDH, subsequent to incubation with secondary antibody. Density of protein band was detected through enhanced chemiluminescence solution (Millipore, Billerica, MA).

### Xenograft tumor assay

6 weeks old male BALB/C nude mice were bought from the National Laboratory Animal Center (Beijing, China) and employed under the approval from the Administrative Panel on Laboratory Animal Care of the Minhang Hospital. Xenograft tumor assay was performed through subcutaneous injection of transfected CC cells to mice. Following 4 weeks of injection, mice were killed before tumors were dissected and weighed.

### Subcellular fractionation

PARIS™ Kit (Ambion, Austin, TX, USA) was acquired for Subcellular fractionation assay in CC cells. The expression levels of FENDRR, U6, GAPDH, were measured by qRT-PCR.

### RNA immunoprecipitation (RIP) assay

EZMagna RIP Kit (Millipore), as well as human antibody against Ago2 or IgG were obtained for RIP assay in CC cells. The precipitated collected by beads were analyzed via qRT-PCR.

### Luciferase reporter assay

Luciferase reporter vectors FENDRR Wt/Mut and TUBA1A Wt/Mut werr generated using the wild-type and mutant miR-15a/b-5p binding sites and pmirGLO Vector (Promega, Madison, WI). After 48 h of co-transfection with indicated plasmids, Dual-Luciferase Reporter Assay System (Promega) was used for luciferase activity.

### RNA pull down

Protein extracts of CC cells were mixed with the Bio-miR-15a-5p or Bio-NC as control in the presence of beads for I h of incubation. The final mixture was subjected to analysis of qRT-PCR.

### Statistical analysis

SPSS 17.0 software (SPSS, Inc., Chicago, IL) was employed in this study for data analysis with Student’s t-test for two groups and one-way or two-way ANOVA for more than two groups, with p < 0.05 as significance. Result from three different repeats was shown as the mean ± standard deviation (SD).

## Results

### FENDRR inhibits CC progression in vitro and vivo

**Fig. 1:**
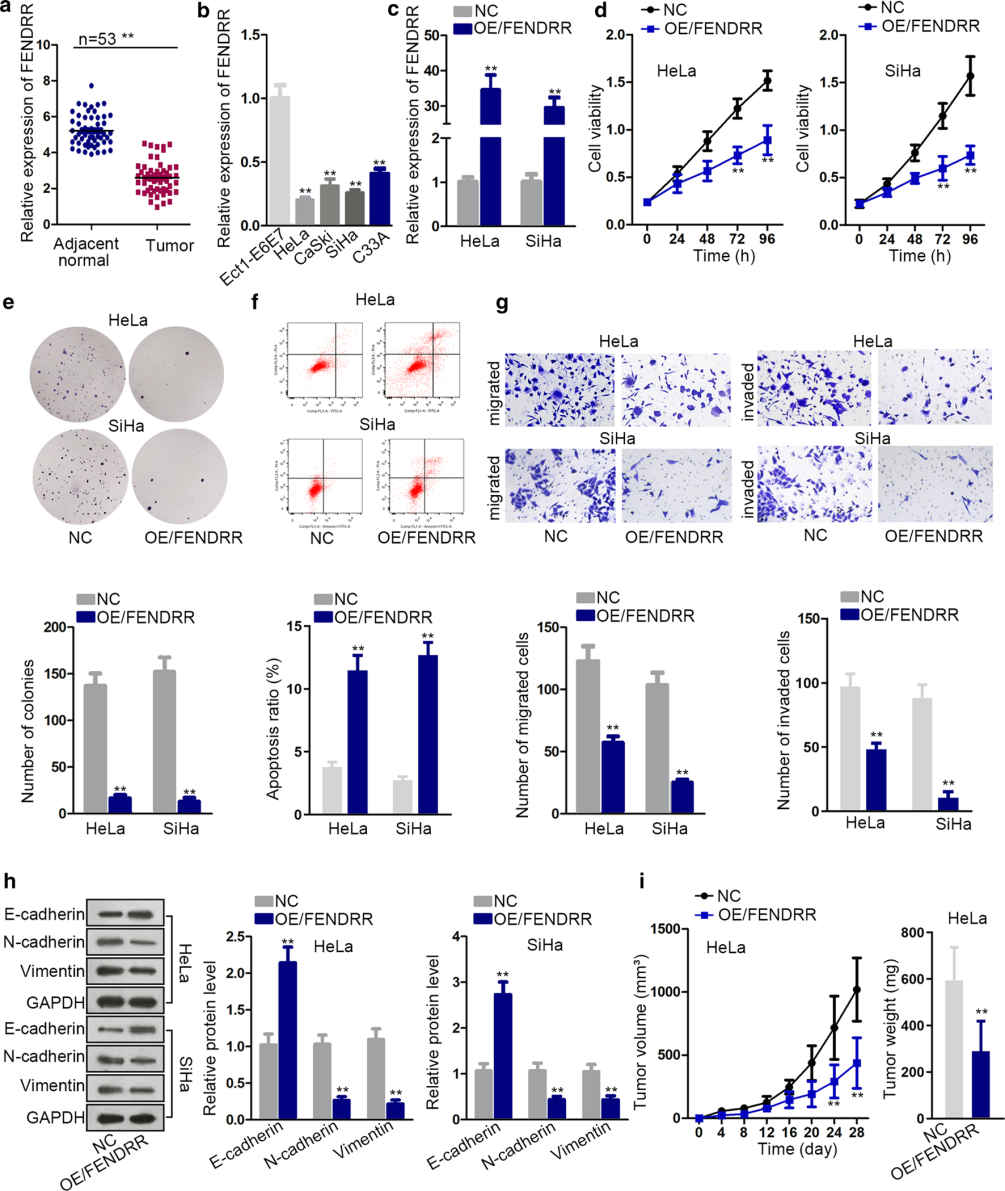
FENDRR inhibits cervical cancer progression in vitro and vivo. **a**, **b** qRT-PCR analysis of relative FENDRR expression in CC tissues and cells. **c** FENDRR overexpression efficiency. **d**–**f** CCK-8, colony formation assay and flow cytometry assay to detect cell viability, proliferation and apoptosis after overexpressing FENDRR. **g** Transwell assays to detect cell migration and invasion after overexpressing FENDRR. **h** Western blot assay examined EMT process after FENDRR overexpression. **i** In vivo experiments demonstrated FENDRR oncogenic role. **p < 0.01

In our study, we first detected relative FENDRR expression in CC tissues and cells via qRT-PCR analysis, finding that FENDRR was significantly reduced in CC tissues and cells (Fig. 1a, b). Next, gain-of-function experiments were carried out to confirm the property of FENDRR in CC. Examination of FENDRR overexpression efficiency was shown in Fig. 1c. Subsequently, cell viability and proliferation ability were tested by CCK-8 and colony formation assay. According to the results, cell viability and proliferation were decreased after overexpression of FENDRR (Fig. 1d, e). Flow cytometry assay demonstrated cell apoptosis ratio increased after FENDRR overexpression (Fig. 1f). Moreover, transwell assays proved that the inhibited cell migration and invasion ability in response to the increased level of FENDRR (Fig. 1g). Western blot detected EMT process slowed down after overexpressing FENDRR (Fig. 1h). Finally, we performed in vivo experiments and observed a decrease in tumor volume and weight after overexpression of FENDRR (Fig. 1i). Taken together, FENDRR functions as a tumor suppressor in CC.

### FENDRR interacts with miR-15a/b-5p

**Fig. 2:**
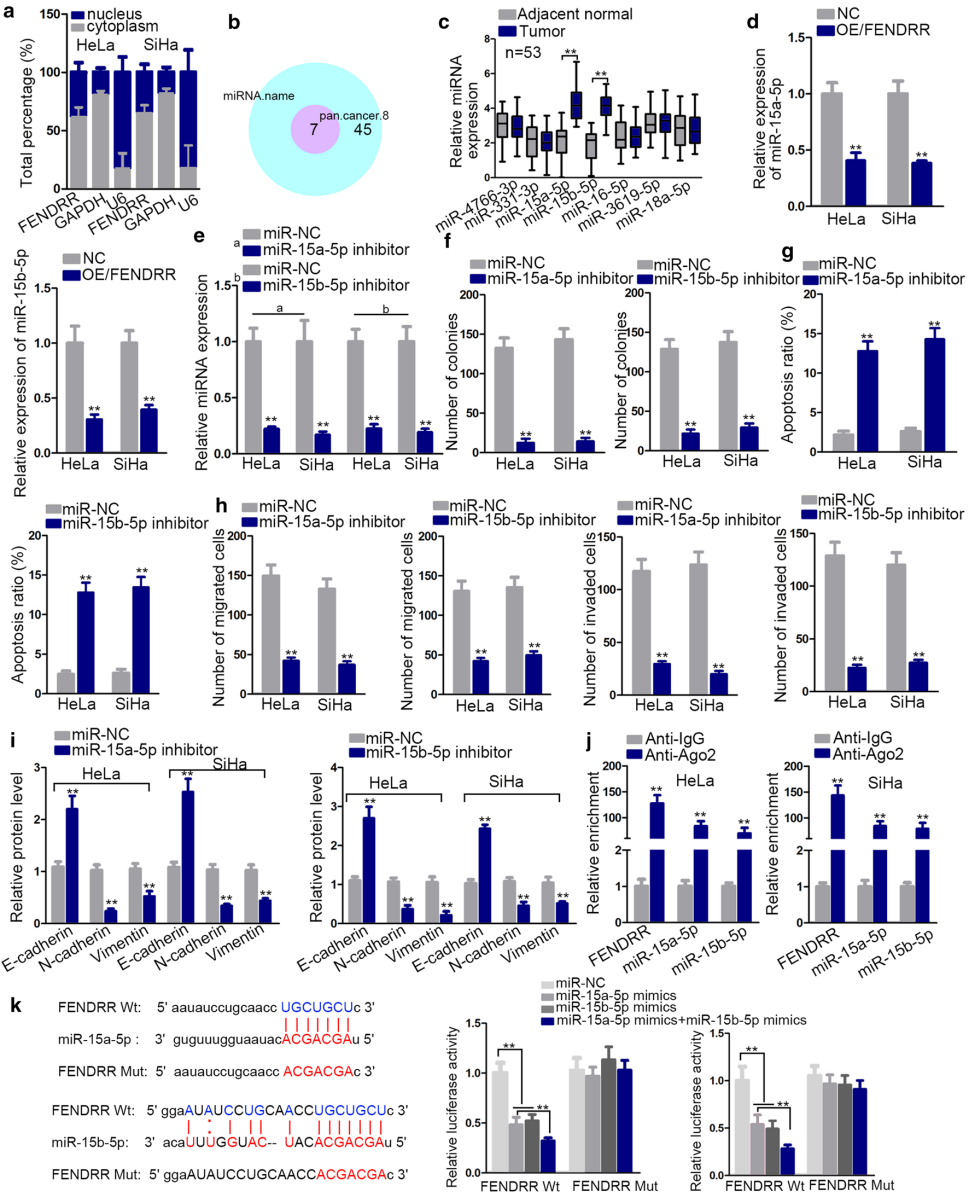
FENDRR interacts with miR-15a/b-5p. **a** Nuclear and cytoplasmic fraction RNA analysis. **b** 7 miRNAs selected with binding sites to FENDRR by informatics analysis. **c** Relative expression of the 7 selected miRNAs in adjacent normal tissues and tumor tissues. **d** MiRNA expression after overexpressing FENDRR. **e** MiR-15a-5p and miR-15b-5p knockdown efficiency test. **f**–**i** Cell proliferation, apoptosis, migration and invasion and EMT process change after knockdown of miR-15a-5p and miR-15b-5p were detected by colony formation assay, flow cytometry assay, transwell assays and western blot. **j** RIP assay verified the coexistence of indicated RNA molecules in RISC. **k** Luciferase reporter assay demonstrated the binding potential between FENDRR and miR-15a/b-5p. **p < 0.01; *n.s.* not significant

To verify the potential mechanism of FENDRR, we analyzed its subcellular location and identified the cytoplasmic location of FENDRR in CC cells (Fig. 2a). By retrieving StarBase database (http://starbase.sysu.edu.cn/), we selected 7 miRNAs sharing binding sites with FENDRR (Fig. 2b). Further qRT-PCR analysis revealed that only miR-15a-5p and miR-15b-5p were upregulated significantly in CC tissues (Fig. 2c). Moreover, the levels of miR-15a/b-5p were decreased by the upregulated FENDRR (Fig. 2d). Next, we applied loss-of-function experiments to confirm the oncogenic property of miR-15a/b-5p. Knockdown efficiency test was conducted at the beginning (Fig. 2e). Through proliferation and apoptosis tests, we confirmed that miR-15a/b-5p silencing had suppressive effect on cell proliferation but accelerated apoptosis (Fig. 2f, g). Similarly, transwell assays verified that the number of migrated or invaded cells was reduced in cells transfected with miR-15a/b-5p inhibitors (Fig. 2h). Additionally, western blot assay detected attenuation in EMT process after miR-15a/b-5p knockdown (Fig. 2i and Additional file 1: Figure S1A). Collectively, we can conclude miR-15a-5p and miR-15b-5p are oncogenes in CC. Furthermore, RIP assay prompted us to conclude that FENDRR, miR-15a-5p and miR-15b-5p coexisted in RNA-induced silencing complex (RISC) (Fig. 2j). By luciferase reporter assay, the interaction between miR-15a/b-5p and FENDRR was further verified (Fig. 2k). Thus, we can confirm FENDRR functions as miR-15a/b-5p sponge.

### FENDRR regulates cervical cancer by modulating miR-15a/b-5p/TUBA1A axis

**Fig. 3:**
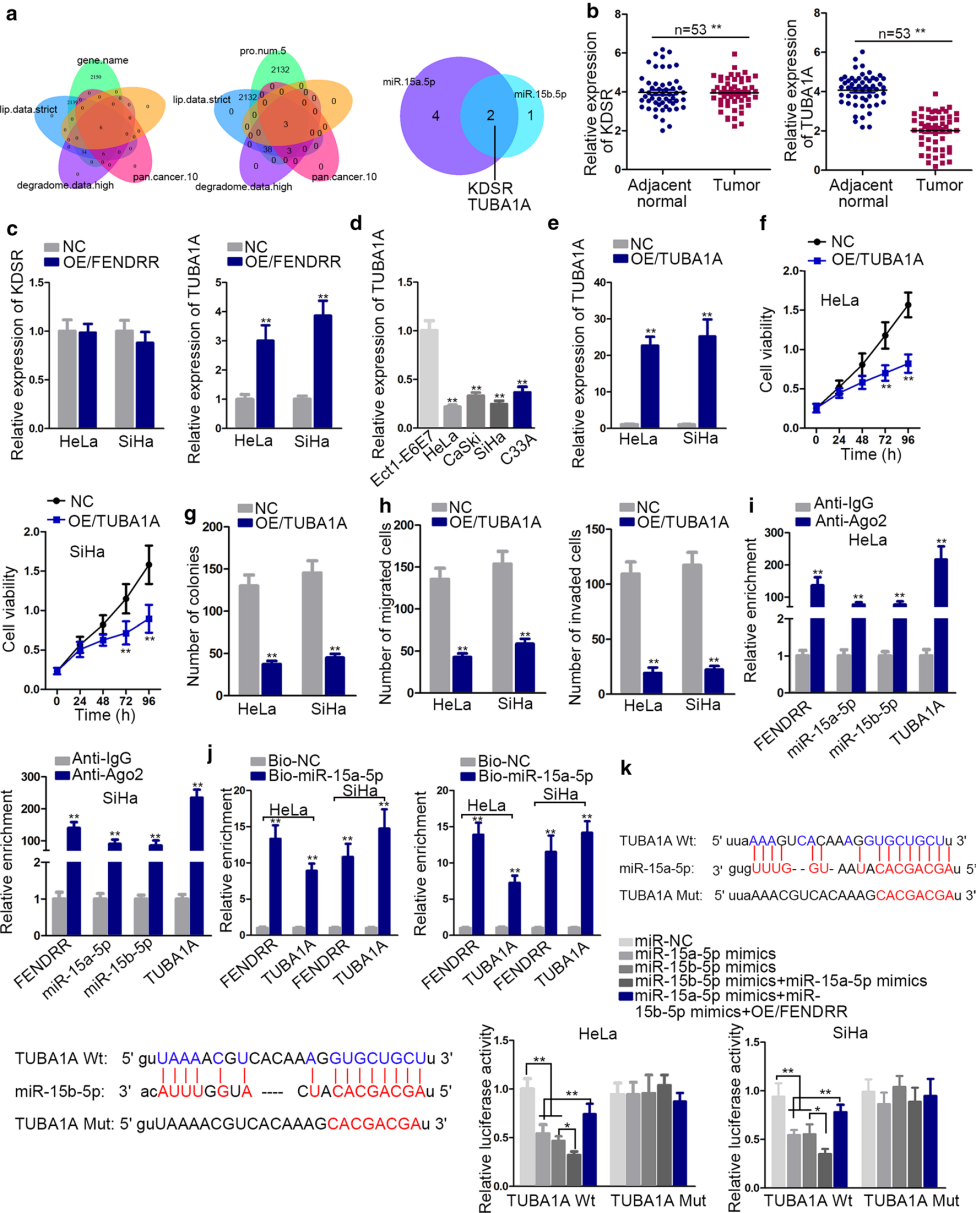
FENDRR regulates CC by modulating miR-15a/b-5p/TUBA1A axis. **a** Bioinformatics analysis of possible mRNAs sharing binding sites with miR-15a/b-5p. **b** The expression of selected mRNA by qRT-PCR. **c** Relevant gene expression after overexpressing FENDRR. **d** TUBA1A relative expression in CC cells. **e** Overexpression efficiency test. **f**, **h** TUBA1A gain-of-function experiments by CCK-8, colony formation assay and transwell assays. **i** Coexistence verification of indicated molecules. **j** Pull down assay detected the PCR product of miR-15a/b-5p. **k** The interaction among FENDRR, miR-15a/b-5p and TUBA1A was demonstrated by luciferase reporter assay. The putative binding sites from StarBase were also verified. **p < 0.01; *n.s.* not significant

Next, to detect the downstream target of miR-15a/b-5p, we resorted to StarBase and found two messenger RNAs (mRNAs) (KDSR and TUBA1A) (Fig. 3a). qRT-PCR examined their relative expression in adjacent normal tissues and tumor tissues, detecting TUBA1A was significantly downregulated in tumor tissues (Fig. 3b). Also, TUBA1A was significantly elevated after overexpression of FENDRR (Fig. 3c). We analyzed TUBA1A expression in CC cells, finding the expression in CC cells is in concert with that in CC tumor tissues (Fig. 3d). Next, we TUBA1A was overexpressed for gain-of-function experiments (Fig. 3e). The subsequent CCK-8 assay and colony formation assay determined the suppressive effect of TUBA1A overexpression on cell viability and proliferation ability (Fig. 3f, g). Meanwhile, migration and invasion ability of CC cells were also attenuated after TUBA1A overexpression (Fig. 3h). Next, mechanistic experiments were applied to verify the competing endogenous RNA (ceRNA) network among the indicated molecules. RIP assay verified TUBA1A, miR-15a/b-5p and FENDRR coexisted in RNA-induced silencing complex (RISC) (Fig. 3i). Pulldown assay confirmed the PCR product of miR-15a/b-5p is TUBA1A and FENDRR (Fig. 3j). Finally, luciferase reporter assay ascertained the interaction among miR-15a/b-5p and TUBA1A and this interaction could be attenuated by FENDRR (Fig. 3k). According to Pearson correlation analysis, FENDRR had positive correlation with TUBAIA in CC tissues (Additional file 2: Figure S2A). In addition, FENDRR and TUBAIA were negatively associated with miR-15a/b-5p (Additional file 2: Figure S2A).

### MiR-15a/b-5p restoration or TUBA1A knockdown reverses the effects of FENDRR silencing on CC cell functions

**Fig. 4:**
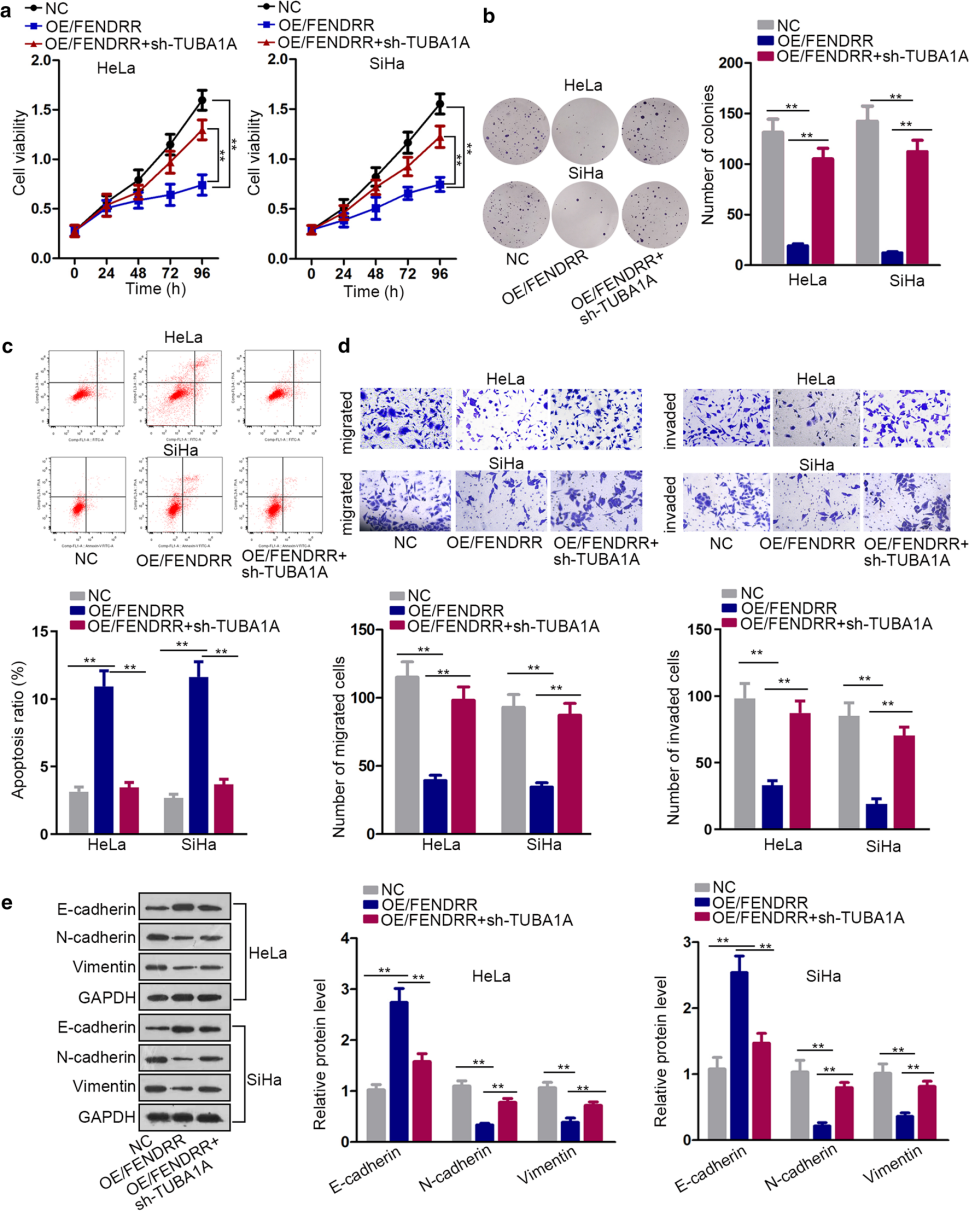
MiR-15a/b-5p restoration or TUBA1A knockdown reverses the effects of FENDRR silencing on CC cell functions. Rescue experiments to test the rescue effects of the rescue group OE/FENDRR + sh-TUBA1A. **a**, **b** CCK-8 assay and colony formation assay showed cell viability and proliferation ability change after the overexpression of FENDRR and transfection with rescue group. **c** Flow cytometry assay proved apoptotic cell number change after overexpressing FENDRR and the treatment of rescue group. **d** Transwell assay demonstrated effects of FENDRR overexpression and transfection with sh-TUBA1A on cell migration and invasion ability. **e** Western blot detected EMT process change by FENDRR overexpression and treatment of the rescue group. **p < 0.01

Finally, we performed rescue experiments to verify whether miR-15a/b-5p and TUBA1A involved in the function depletion caused by FENDRR overexpression. CCK-8 and colony formation assay proved that cell viability and proliferation ability decreased by FENDRR overexpression were completely recovered after knockdown of TUBA1A (Fig. 4a, b). Flow cytometry assay showed the apoptosis ratio increased in FENDRR-upregulated CC cells was reduced again after knockdown of TUBA1A (Fig. 4c). Furthermore, cell migration, invasion and EMT process were observed in FENDRR-overexpressed CC cells after silencing of TUBA1A. As depicted in Fig. 4d, e, the attenuation of cell migration and invasion ability by FENDRR overexpression was abolished after suppression of TUBA1A expression. Additionally, rescue experiments with overexpressed miR-15a-5p and miR-15b-5p were carried out. According to Additional file 2: Figure S2B–E and Additional file 3: Figure S3A, cellular processes, including cell viability, proliferation, apoptosis, migration, invasion and EMT process affected by FENDRR overexpression were partly rescued by the upregulation of miR-15a-5p or miR-15b-5p alone but was completely rescued by the co-overexpression of miR-15a-5p and miR-15b-5p. Taken together, we conclude that FENDRR inhibits CC progression by modulating miR-15a/b-5p/TUBA1A axis.

## Discussion

Recently, more and more lncRNAs have been identified dysregulated in CC and regulating CC progression including XLOC_006390, highly upregulated in liver cancer (HULC), Growth arrest-specific transcript 5 (GAS5), Small nucleolar RNA host gene 20 (SNHG20), etc. [15–18]. We found lncRNA FENDRR hasn’t been investigated in CC to date. Also, FENDRR was reported inhibiting various cancers such as colon cancer, hepatocellular carcinoma, etc. [13, 19]. Similarly, FENDRR also had tumor suppressive property in CC, which was reflected by its downregulation in CC tissues and cells as well as the results of gain-of-function experiments.

Increasing studies have revealed that lncRNAs function as a ceRNA to sequester microRNAs (miRNAs) and thus modulate the downstream mRNAs [20, 21]. For instance, lncRNA Plasmacytoma variant translocation 1 (PVT1) facilitates glioma by targeting miR-128-3p/Gremlin-1 (GREM1) axis [22]. LncRNA Interleukin enhancer-binding factor 3 antisense RNA 1 (ILF3-AS1) accelates melanoma progression via miR-200b/a/429 axis [23]. In our study, by means of StarBase, we found relevant miRNAs having binding sites with FENDRR. By examining the relative expression in tumor tissues and their adjacent normal tissues, we discovered miR-15a-5p and miR-15b-5p was prominently upregulated in tumor tissues. MiR-15a-5p and miR-15b-5p have been previously reported as promoters in diseases such as abdominal aortic aneurysm, osteoarthritis chondrocytes and colorectal adenocarcinoma [24–26]. For further evidence, miR-15a/b-5p loss-of-function experiments were carried out and the oncogenic property of miR-15a/b-5p was confirmed. Next, the RIP assay proved miR-15a/b-5p and FENDRR coexisted in RISC and from pull down assay, we can observe the PCR product of miR-15a/b-5p is FENDRR, indicating that miR-15a/b-5p bind to FENDRR. Furthermore, luciferase reporter assay verified the interaction between miR-15a/b-5p and FENDRR. To select the downstream target gene of miR-15a/b-5p, we resorted to StarBase again and two mRNAs were finally selected. After testing the relative expression in CC tissues and their respective reaction to FENDRR overexpression, we confirmed TUBA1A was qualified target gene of miR-15a/b-5p. Studies have investigated the involvement of TUBA1A in ceRNA network. So next step, we applied gain-of-function experiments and observed cell viability, proliferation, migration and invasion ability decreased after overexpression of TUBA1A. Mechanism experiments further elucidated the ceRNA network constituted by FENDRR, miR-15a/b-5p and TUBA1A. Finally, the rescue experiments further demonstrated that knockdown of TUBA1A saved the function depletion caused by FENDRR overexpression.

## Conclusion

Our study is the first to investigate the mechanism and biological effects of FENDRR in CC and we studied functions of TUBA1A as the ceRNA in CC. The identification of FENDRR and TUBA1A provides us novel biomarkers for diagnosis and therapy of CC and the FENDRR/miR-15a/b-5p/TUBA1A axis opened up a new insight in targeted mechanism in CC. Also, more investigation on TUBA1A functions will be continued.

## Supplementary information

**Additional file 1: Figure S1.** A. Proteins involved in EMT process were detected in CC cells transfected with the inhibitor of miR-15a-5p or miR-15b-5p. **p < 0.01.

**Additional file 2: Figure S2.** A. Pearson correlation analysis of the relationship among FENDRR, miR-15a/b-5p and TUBA1A in CC tissues. B–E. Rescue experiments detected variation in cell viability, proliferation, apoptosis, migration and invasion with the treatment of overexpressed miR-15a/b-5p by CCK-8, colony formation assay, flow cytometry assay and transwell assays. *p < 0.05; **p < 0.01.

**Additional file 3: Figure S3.** A. Proteins involved in EMT process were detected in treated CC cells. *p < 0.05; **p < 0.01.

## Data Availability

Research data and materials are not shared.

## Abbreviation

LncRNAs: Long non-coding RNAs
CC: Cervical cancer
ceRNA: Competing endogenous RNA
mRNAs: Messenger RNAs
miRNAs: MicroRNAs
FENDRRF: Fetal-lethal non-coding developmental regulatory RNA
miR-15a-5p: MicroRNA-15a-5p
miR-15b-5p: MicroRNA-15b-5p
TUBA1A: Tubulin alpha1A
qRT-PCR: Quantitative real-time polymerase chain reaction
RIP: RNA immunoprecipitation
EMT: Epithelial–mesenchymal transition
FBS: Fetal bovine serum
DMEM: Dulbecco’s modified eagle medium
ATCC: American type culture collection
cDNA: Complementary DNA
NC: Negative control
CCK-8: Cell counting kit 8
SDS-PAGE: Sodium dodecyl sulfate polyacrylamide gel electrophoresis
PVDF: Polyvinylidene fluoride
U6: U6 small nuclear RNA
GAPDH: Glyceraldehyde-3-phosphate dehydrogenase
RISC: RNA-induced silencing complex
SD: Standard deviation
MEG3: Maternally expressed gene 3
NEAT1: Nuclear paraspeckle assembly transcript 1
SOX4: Sry‑related HMG‑BOX‑4
RUNX1: Runt-related transcription factor 1
HULC: Highly upregulated in liver cancer
GAS5: Growth arrest-specific transcript 5
SNHG20: Small nucleolar RNA host gene 20
PVT1: Plasmacytoma variant translocation 1
GREM1: Gremlin-1
ILF3-AS1: Interleukin enhancer-binding factor 3 antisense RNA 1
