# Supplementary figures and images for: FENDRR suppresses cervical cancer proliferation and invasion by targeting miR-15a/b-5p and regulating TUBA1A expression
Source: Cancer Cell Int. 2020 May 6;20:152. doi: 10.1186/s12935-020-01223-w (PMC7204253; doi:10.1186/s12935-020-01223-w)

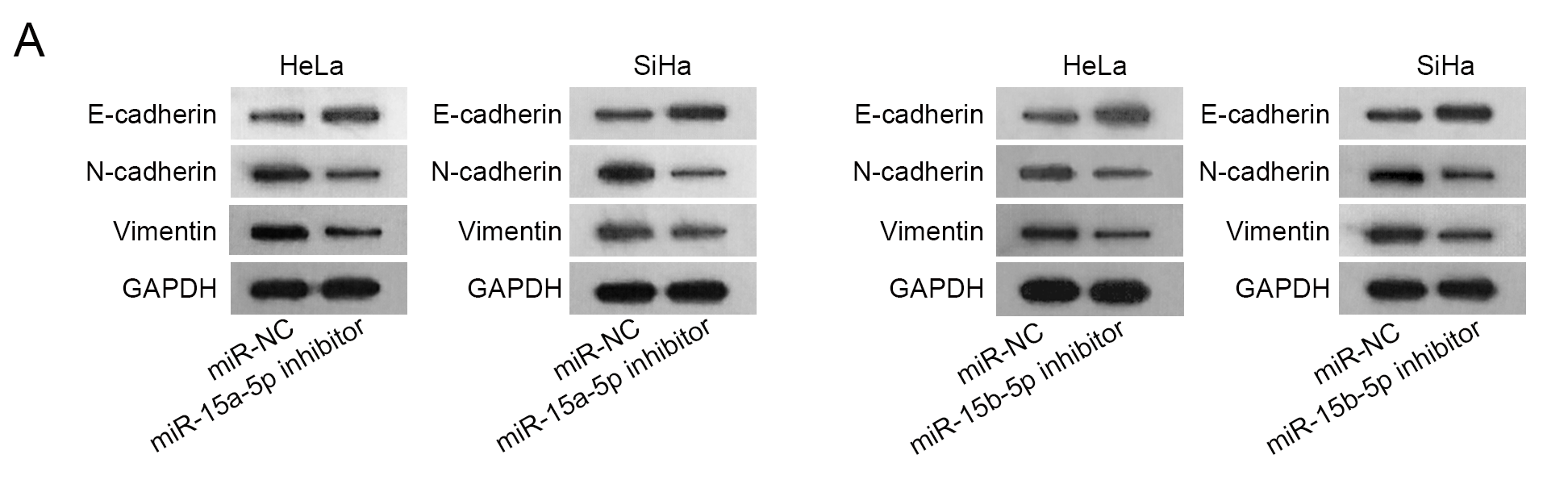

Supplement: Supplementary file 1 — Additional file 1: Figure S1. A. Proteins involved in EMT process were detected in CC cells transfected with the inhibitor of miR-15a-5p or miR-15b-5p. **p < 0.01. [file 12935_2020_1223_MOESM1_ESM.tif]

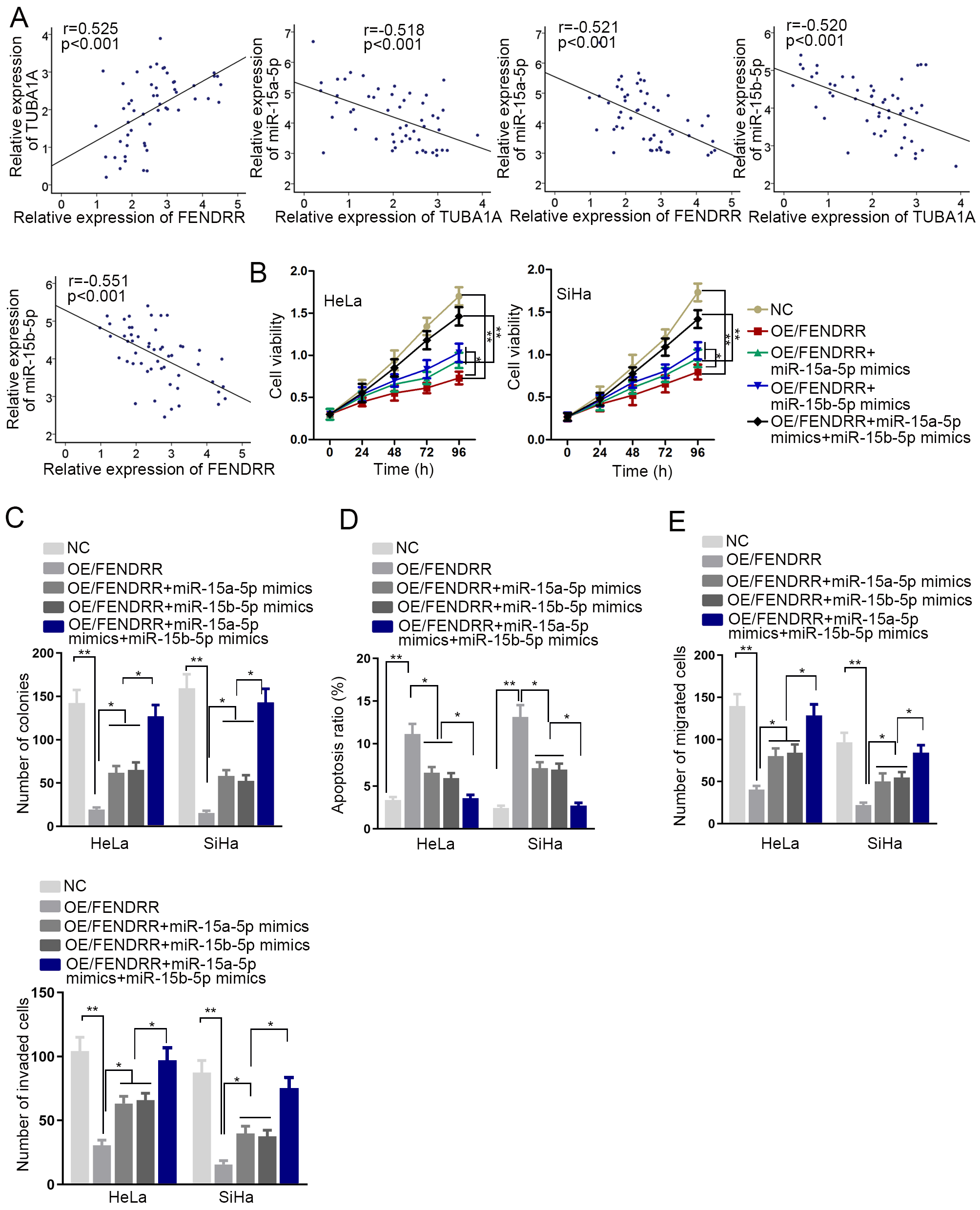

Supplement: Supplementary file 2 — Additional file 2: Figure S2. A. Pearson correlation analysis of the relationship among FENDRR, miR-15a/b-5p and TUBA1A in CC tissues. B–E. Rescue experiments detected variation in cell viability, proliferation, apoptosis, migration and invasion with the treatment of overexpressed miR-15a/b-5p by CCK-8, colony formation assay, flow cytometry assay and transwell assays. *p < 0.05; **p < 0.01. [file 12935_2020_1223_MOESM2_ESM.tif]

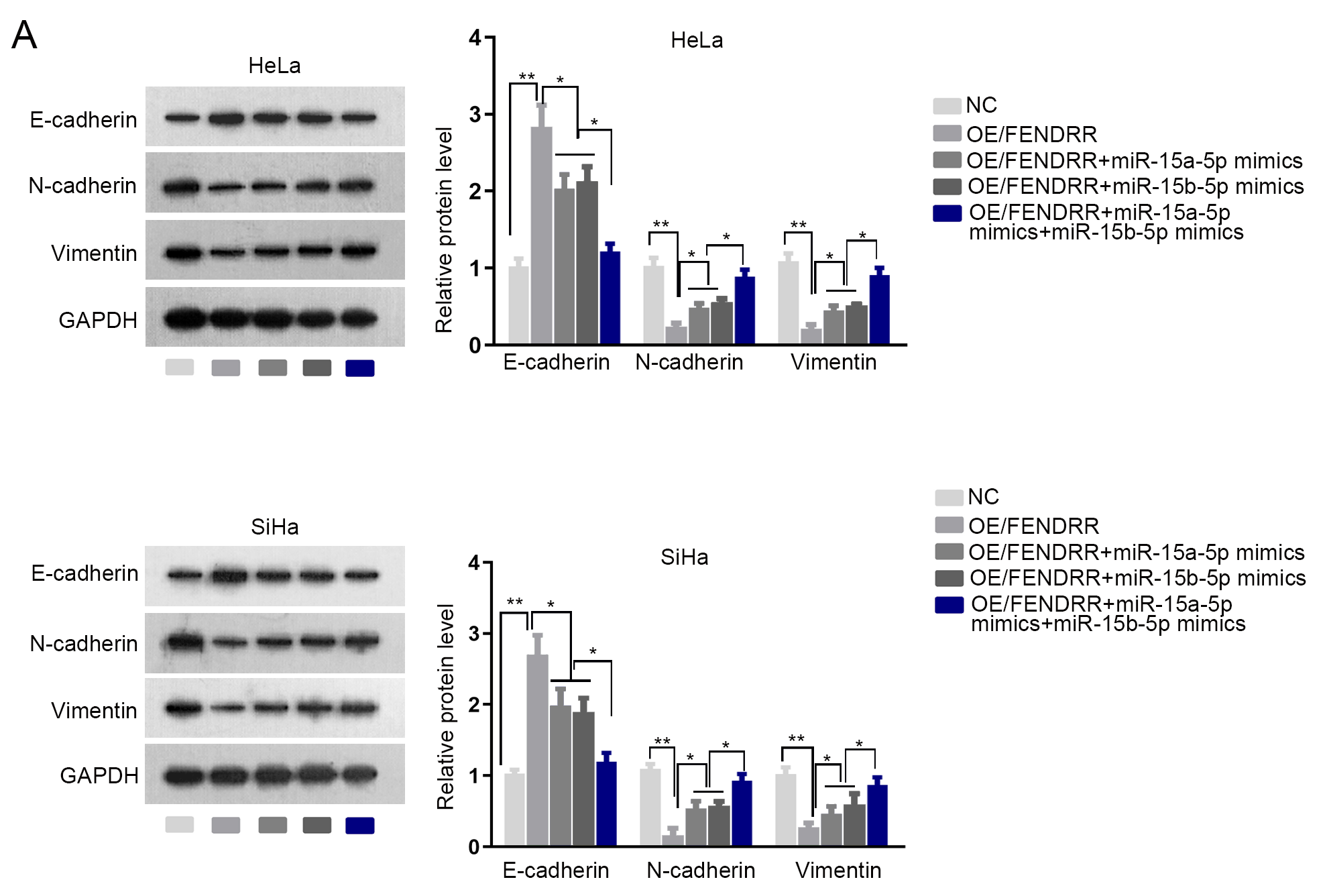

Supplement: Supplementary file 3 — Additional file 3: Figure S3. A. Proteins involved in EMT process were detected in treated CC cells. *p < 0.05; **p < 0.01. [file 12935_2020_1223_MOESM3_ESM.tif]
